# Pennsylvania Association of Dental Surgeons

**Published:** 1864-08

**Authors:** James Truman


### Proceedings of Societies.

### PENNSYLVANIA ASSOCIATION OF DENTAL SURGEONS.

Reported by JAMES TRUMAN, D. D. S.

At a monthly meeting of the Association held July 21st, at 8 o'clock—

The subject for the evenings discussion "materials for filling teeth," continued from the last meeting, was opened by Dr. Buckingham, who said he had a new idea suggested to him by a member of the profession in regard to the preparation of gold for filling teeth. This was to plane it off in shavings instead of beating it out as gold foil is made. The gentleman's attention had been called to it by experiments made with tin turnings, which led him to continue the tests in gold, which worked equally well.

The Doctor thought if gold could be planed sufficiently thin it might be worked this way. It probably could not be made into large sheets like gold foil, but it could be shaved off into ribbons, a quarter of an inch wide, and these could be used without folding. He thought there might be some advantage in preparing gold in this way. The surface of the gold would be different from that which had been beaten, and he thought more adhesive. If gold planed off in shavings, could be used as well as gold foil, it would be a great saving to dentists, as the labor expended upon foil enhances its value very much.

We all know that freshly cut surfaces of lead will adhere, but if exposed to the air for sometime they loose this property. Platina precipitated from a solution, will weld if heated to a full red color and then hammered; but if the spongy mass has been burnished in any place, before heating, it will not weld at that spot. This shows that the atoms can not be restored to their original condition by heating, and he thought it probable that the atoms of gold when being beaten into foil, would, in a measure, loose the property of welding.

Another member of the profession, suggested to him a process that he had used of annealing gold foil. He placed it in boiling water and dried it in an oven. Whether this would have the desired effect the Doctor could not tell; but all suggestions that aimed to improve our operations were worthy of attention and experiment.

Dr. Hayhurst thought gold prepared after the manner suggested by Dr. Buckingham might possibly work well when freshly cut, but thought any keeping on hand would destroy the adhesive power. It is well known that a bullet cut in half will adhere closely if united at once; but if allowed to remain for some time will not. He had tried gold in every conceivable way, but had not been as successful as some members of the profession represented they had been. He thought his trouble was owing to imperfect instruments. He had no doubt but that it was the best material to use.

His experience was limited in the use of tin. Oxichloride he had used to some extent, and had found it would last from six months to a year, Amalgam when well put in will last an almost unlimited period. He did not think it was always in the material or manipulation, that would preserve the teeth.

Dr. Truman said the discussion had taken a somewhat different course from what he had hoped it would, as he was desirious of hearing opinions in regard to the best modes of using the materials as we now have them.

There were, in his judgment, four primary considerations that were absolutely essential to perfect manipulacion of the metals in filling:

1. Proper selection of material.
2. Instruments properly shaped and serrated.
3. Perfect consolidation as the operation proceeds.
4. Freedom from moisture.

Gold occupying the pre-eminent position that it does and should, it is important that we should get the best that is offered in the market. From some cause, difficult for the uninitiated to explain, there is great difference in the manner of working foil of different manufacturers. Each one must select that which his experience has decided as the best. The Doctor prefers that manufactured by Mr. S. S. White, as he has found it to possess properties that peculiarly fit it for the wants of the operator; but this, like all other foils, if kept on hand too long, will loose its adhesive character to a great extent.

The cause of the failures in filling teeth, may, in a great measure, be attributed to a want of properly made instruments. The serrations must be clean and sharp and kept so. The ragged condition in which they are often left by the manufacturer, can be removed by the use of emery upon a soft pine board, by passing it quickly back and forth, the serrations will imbed themselves in the wood and become exposed to the action of the emery at all points. As this part of the instrument is the most delicate, the points are liable to fracture. This must be constantly watched and remedied, for it is an impossibility to make a filling, when the gold or tin will present a perfectly solid mass, through all parts of the cavity, unless the serrations are constantly kept in perfect condition. Fillings can be made by the use of cylinders and pellets, of various forms, presenting externally a solid surface, without the use of serrations, but the metal always retains the individualised character in which it is inserted, and is necessarily imperfect.

The mistake most frequently made by the young operator, and by some who claim extensive experience, is a defect in consolidating the material from beginning to the end of the operation. The idea that it is necessary to allow the metal to retain a degree of its original soft character, is fatal to perfection. The more thoroughly the foil is condensed, the more readily subsequent portions can be welded to it. It is impossible to get gold or tin so completely consolidated that the folds will not weld with proper instruments, provided the surface is left rough and dry. To accomplish this perfectly the mallet is used to consolidate from the beginning of the operation.

To secure freedom from moisture, a free use of napkins is necessary, arranged to completely defend the tooth to be filled from the inroad of saliva.

Tin foil requires somewhat different treatment from gold. the manufacturers give it to us prepared in sheets of various thickness. The rule to be observed, is to cut the sheet of tin into strips of a width proportionate to the thickness used. The thicker it is, the narrower the strip. If folded lengthwise in the rope form, it must be done as gently as possible, one or two fold upon itself is all that the metal will bear. To roll it tightly will destroy its adhesive character. The heavy article welds better by cutting it into narrow strips the width of the cavity and packing it without folding. Although the form of using the tin is not of material consequence provided that the metal be allowed to retain as much as possible its original condition, yet the Doctor had found the most convenient mode for him was to fold it over a narrow spatula shaped instrument, made from the main spring of a watch. By placing the strip to be folded upon the palm of the hand, it could be brought into the shape of a narrow piece of tape, without much disturbance of the metal. He then cuts it into convenient lengths.

The Doctor thought both gold and tin could be made as solid as the density of the metals would allow. In illustration of which, he exhibited gold fillings brought into plate: and a model of a bicuspid tooth built up with tin foil, upon a plate of block tin. The tin foil tooth was apparently of equal density to the block tin upon which it was built. Though the operation was performed out of the mouth, his experience led him to believe it could be as well done in the mouth. Tin is less affected by moisture than gold, and packs readily under water. As a preservative against the inroads of caries, the Doctor considered tin equal to gold, when not upon the masticating surface, its density not being sufficient to resist for a series of years the abrasion of the molar teeth.

Dr. West could endorse the opinion that had been expressed favorable to tin foil. He thought in sensitive teeth it was better than gold, it being a poor conductor of heat; but for general purposes he believed gold superior to all materials in use. He would use amalgam only in peculiar cases.

Dr. H. Townsend said that though he was an advocate for gold in all cases where it could be used, believed there were some teeth that could be preserved with amalgam, when gold could not be used. He had never seen a case where he could trace constitutional effects from its use. He had one patient in whose mouth he placed fourteen amalgam fillings. The cavities were very sensitive and very shallow; and the lady being of a very nervous temperament, could not bear the least pressure upon the teeth without severe pain. Her health had been poor for sometime previous, with no appetite. Her teeth were carefully cleaned and filled, and five roots removed under the influence of either. In less than two months her health was greatly improved, with a constantly increasing appetite. In five or six months heard from her again and she was gaining in flesh and strength.

He considered it very important to prepare all cavities with great care, no matter what material is used for filling. He thought many teeth had been ruined by the injudicious use of amalgam.

Dr. Barker was of the opinion that no one material could be used exclusively; but considered that gold foil was superior to any other in the great majority of cases. Special cases will present, when some plastic material must be used or the teeth removed. In some cases when the cavity can not be kept dry, tin foil filling can be made that will answer the purpose better than anything else. In others where there is an absence of a considerable portion of the crown, or there are weak walls, good fillings can be made of Wood's Plastic Metallic Filling or Amalgam. The first named material he liked better and better the more he used it and became profficient in manipulating it. He had recently examined several fillings introduced some months since which looked as perfect as when first filled.

Amalgam was an article which also had an appropriate place and might, he considered, be used in cases where nothing else would accomplish so good and perfect a result. An instance of this had happened that very day in his practice and he would detail it. The cavity was in the buccal surface of the upper third molar, right side, in the mouth of a gentleman weighing at least 250, with cheeks so fleshy that no view of any consequence could be obtained of the cavity. It could not be kept dry, and no other material but amalgam or perhaps oxichloride (which they considered worthless) could have been introduced. He succeeded in making a good filling and believed it would preserve the tooth. Amalgam had to stand an amount of abuse which it did not deserve. In consequence of its being plastic, care was not taken to form the cavity properly, and too many were in the habit of pressing in a large portion at a time, so that the amalgam was not in perfect contact with the walls. Again it was generally used in teeth considerably diseased and decayed, and if inflammatory symptoms showed themselves it was erroneously attributed to the materal. He considered the very last influence exerted by the mercury of the amalgam (admitting for the sake of argument there is an influence) would be upon the periosteum of the tooth. It might be barely possible that enough mercury could be eliminated by amalgam plugs to exert a systemic influence, though he did not believe it, unless it might be in some rare case of idiosyncrasy; but even in such cases the mercury would have to be absorbed into the circulation and then if ptyalism was induced the gums and periosteum might be affected, but no such local affection as periostitus or alveolar abscess would result. In several cases of ptyalism which had come under his notice, he could not rememember a single instance where the pulps of teeth had died from the influence of the disease; and he had yet to see a case of alveolar abscess on a tooth with a living pulp.

The integrity of amalgam fillings depend much upon the preparation of the material. Some were in the habit of mixing the amalgam in a morter, afterwards pressing the free mercury out with the thumb; others would first wash out the oxides of the metals with alcohol. A much better way was the method advocated by a member of this association and published in one of the former reports. His mode of proceedure was to combine twice the quantity of common salt to the quantity of amalgam and mercury, rubbing them well together for some time. The salt will become quite dark, and the first drops of water added will be turned black, proving that the oxides have been removed. It should be thoroughly washed to remove the salt, then placed in a chamois skin and pressed with *considerable force* to remove all the mercury possible. When taken from the skin it will be very solid, but by holding it in the hand for a few moments, it will become soft enough to work well. It can then be used in small pieces, like sponge gold, packing piece after piece, until the cavity is full. The advantage of this mode of proceedure was, that amalgam fillings in many cases retained their silver color, the tooth not becoming discolored. here was also less danger of the elimination of free mercury.

Dr. Buckingham had used amalgam at times, but his experience had been that it generally became defective at the edges, produced by the oxidation of the metal at that point. He was not aware that a perfect case of ptyalism had ever been reported arising from its use. He thought if there was any free mercury escaping from the filling, that it would pass off without producing any effect.

He had but little faith in bone filling. Confined his practice to gold and tin. He had found advantage in using heavy tin without folding, cutting it into narrow strips. The thicker you have your gold, the longer you must have the serrations of your instruments. In answer to a suggestion of Dr. Griffith, thus if gold was packed solid in the centre it drew from the circumference, said he thought the Doctor's trouble originated in not packing his gold solid at the bottom of the cavity. If this was done, it could not draw from the circumference to the centre. The tendency in a perfectly solid filling, is to expand. In answer to a question whether he thought mercury could be extracted from an amalgam filling after long wear, said he thought it could. There could be no change by time.

The subject " Influence of Diseased teeth upon the health." was chosen for discussion at the next monthly meeting in September. Adjourned.

